# The Best Antiseptic

**Published:** 1896-03

**Authors:** 


### The Best Antiseptic.

Dr. J. B. Walker, of Indianapolis, Ind., after several years' use of Listerine, says he considers it the best antiseptic and prophylactic we have.

When patients complain almost continuously of toothache or sensitive teeth, it is usually an indication to administer the phosphate of lime.

